# Characterization and Immune Function of *NOD1* in Snakehead (*Channa argus*)

**DOI:** 10.3390/biology15120942

**Published:** 2026-06-16

**Authors:** Beibei Wang, Yiying Liu, Xiaochen Zhu, Min Cao, Qiang Fu, Yang Li, Ning Yang, Xiaoyan Zhang, Guangzhou Wu, Chao Li

**Affiliations:** 1 School of Marine Science and Engineering, Qingdao Agricultural University, Qingdao 266109, Chinaqiangfu@qau.edu.cn (Q.F.);; 2 Shandong Qidu Pharmaceutical Co., Ltd., Zibo 255400, China; 3 Jining Fishery Development and Resource Conservation Center, Jining 272000, China

**Keywords:** *Channa argus*, *NOD1*, NF-κB, *RIPK2*, innate immunity

## Abstract

The innate immune response is a critical defense mechanism by which vertebrates recognize and eliminate invading pathogens. Pattern recognition receptors (PRRs) detect pathogen-associated molecular patterns and activate downstream signaling pathways. *NOD1*, a classic PRR of the NLR family, recruits the adaptor protein *RIPK2* to initiate antibacterial signaling. In this study, we cloned and characterized the *NOD1* gene from snakehead (*Channa argus*). Briefly, the full-length *NOD1* cDNA is 2829 bp encoding 943 amino acids, showing high homology with Perciformes. The qPCR analysis revealed widespread *NOD1* gene expression in various tissues, with significant upregulation in the gill (*p* < 0.05) and spleen (*p* < 0.05) following bacterial infection. Overexpression of the *NOD1* gene activated the NF-κB signaling pathway in a dose- and time-dependent manner, and specifically responded to the bacterial ligand iE-DAP but not to other tested ligands. Furthermore, *NOD1* synergized with the downstream adaptor *RIPK2* to enhance NF-κB activity, and direct protein interaction between *NOD1* and *RIPK2* was confirmed by co-immunoprecipitation. Taken together, these findings demonstrate that snakehead *NOD1* plays a critical role in the host antimicrobial immune response.

## Simple Summary

As the most representative member of the NLR family of pattern recognition receptors, *NOD1* plays a crucial role in various biological processes, including innate and adaptive immunity in vertebrates. This study primarily aimed to characterize the role and response mechanisms of *NOD1* in the innate immune response of snakehead. The *NOD1* transcript was highly expressed in the mucosal immune tissues of snakehead, and its overexpression significantly induces NF-κB activity. Further studies revealed that *NOD1* specifically recognizes iE-DAP and acts synergistically with the *RIPK2* gene to enhance NF-κB promoter activity. Together, these findings reveal that *NOD1* plays a vital role in the fish’s natural defense system. Understanding this process can help scientists develop better strategies to prevent disease outbreaks in farmed fish, reducing the need for antibiotics and supporting sustainable aquaculture.

## 1. Introduction

Innate immunity serves as the first line of defense against invading pathogens, initiating with the recognition of pathogen-associated molecular patterns (PAMPs) by pattern recognition receptor (PRR) [1]. Among various PRR families, the NOD-like receptor (NLR) family is one of the largest in terms of the number of genes encoded in vertebrate genomes [2,3]. NLR proteins can sense a variety of pathogenic and non-pathogenic stimuli, thereby inducing distinct signal transduction mechanisms [4]. For instance, members of the NLR family are capable of activating multiple innate immune pathways, including nuclear factor-κB (NF-κB) signaling and cytokine production, as well as inducing innate immune cell death [5]. NLRs are involved in two main processes: firstly, they act as regulators of inflammation, and secondly, they form multi-protein inflammasome complexes. The formation of these complexes triggers the proteolytic cleavage of caspase-1 and induces pyroptosis [6,7].

The structural characteristics of NLRs are pivotal in determining their specificity and the extent of their activity, consisting of three core domains: an N-terminal variable domain for downstream signal transduction, a central nucleotide-binding domain (NBD) (also known as the NACHT domain) for oligomerization, and a C-terminal leucine-rich repeat (LRR) domain [8,9]. The LRR domain can sense PAMPs and damage-associated molecular patterns (DAMPs) in host cells to initiate innate immune responses [5]. Based on the characteristics of additional unique domains, NLRs are classified into five categories: NLRA, NLRB, NLRC, NLRP, and NLRX. Among them, NLRC1 contains a caspase recruitment domain (CARD) at the N-terminal region and is also known as *NOD1* [10]. Specifically, as an intracellular pattern recognition receptor, *NOD1* plays a critical role in antibacterial immunity by recognizing the bacterial peptidoglycan motif iE-DAP within the host cytosol after pathogens or their components gain access to the intracellular compartment, and binds to RIP2 through its CARD, thereby activating the downstream NF-κB and MAPK pathways [11].

The *NOD1*-RIP2 axis and its domains are conserved in teleosts [12,13], such as zebrafish (*Danio rerio*) [14,15], goldfish (*Carassius auratus* L.) [16,17], rainbow trout (*Oncorhynchus mykiss*) [18], sturgeon (*Acipenser ruthenus*) [19] and Chinese perch (*Siniperca chuatsi*) [20]. Snakehead (*Channa argus*) is an economically important freshwater fish species widely cultured in East Asia, especially in China. However, intensive farming has made it highly susceptible to bacterial diseases, among which *Nocardia seriolae* infection is the most prevalent [21]. *N. seriolae* is the primary pathogen causing nocardiosis in various fish species, leading to significant economic losses in the aquaculture industry [22,23]. Naturally infected snakehead exhibit typical clinical signs, including slow swimming and reduced food intake, skin wounds, anal swelling, ascites, and white granulomatous lesions in the liver, spleen, and kidney [24]. Gross pathological examination reveals numerous ivory-white nodules (1–5 mm in diameter) scattered on multiple internal organs, accompanied by visceral tissue swelling and hemorrhage. Histopathological examination shows typical systemic granuloma formation with well-defined granulomatous structures and extensive cellular infiltration [25]. Pathogenicity testing has demonstrated that *N. seriolae* infection in snakehead results in mortality rates ranging from 70% to 100% [22]. *N. seriolae* was selected as the representative pathogen in this study for several reasons. First, this bacterium is the primary etiological agent of nocardiosis in a wide range of economically important marine and freshwater fish species. It has been reported to infect approximately 42 species of marine and freshwater fish worldwide, including largemouth bass (*Micropterus salmoides*), spotted sea bass (*Lateolabrax maculatus*), large yellow croaker (*Larimichthys crocea*), Chinese rice-field eel (*Monopterus albus*), and snakehead [22,26], thereby causing substantial economic losses to the aquaculture industry [26]. Second, in snakehead, nocardiosis is one of the most prevalent and devastating bacterial diseases under intensive farming conditions, and *N. seriolae* infection in this species has been shown to induce mortality rates [22]. Third, *N. seriolae* has a long incubation period, a slow but progressive infection course, and a strong propensity to establish chronic granulomatous infections in host tissues [23], making it an excellent model for investigating host antibacterial immune mechanisms, particularly those involving intracellular pattern recognition receptors. Importantly, *N. seriolae* is a facultative intracellular parasite that can survive within macrophages, and *NOD1* is also intracellular. Therefore, *N. seriolae* infection is particularly suitable for studying *NOD1*-mediated recognition and signaling. Furthermore, *N. seriolae* possesses the molecular basis for *NOD1* recognition. As a Gram-positive actinobacterium belonging to the order Mycobacteriales, *N. seriolae* has a complex, multi-layered cell wall that contains abundant peptidoglycan as a structural component [27]. The peptidoglycan of Nocardia species, including *N. seriolae*, contains meso-diaminopimelic acid (meso-DAP) as a key constituent of the tetrapeptide side chain [28,29]. Meso-DAP is an essential component of bacterial peptidoglycan and serves as the direct structural precursor of γ-D-glutamyl-meso-diaminopimelic acid (iE-DAP), the minimal bioactive motif specifically recognized by *NOD1* [30,31]. Furthermore, genomic analysis of *N. seriolae* has identified the complete set of genes required for peptidoglycan biosynthesis, including murE-2 (UDP-N-acetylmuramoyl-L-alanyl-D-glutamate-2,6-diaminopimelate ligase), responsible for adding meso-DAP to the peptidoglycan precursor, and lysA (diaminopimelate decarboxylase), involved in meso-DAP metabolism [32]. Thus, *N. seriolae* naturally produces the *NOD1* ligand iE-DAP as an integral component of its cell wall peptidoglycan. This provides a direct molecular link between the pathogen and the *NOD1* signaling pathway, justifying the use of *N. seriolae* infection as a relevant in vivo model to study *NOD1*-mediated antibacterial immune responses in snakehead. Given that *NOD1* is a key intracellular pattern recognition receptor involved in antibacterial immunity, we selected *N. seriolae* as the representative pathogen to investigate the immune function of *NOD1* in snakehead. Despite its commercial importance, the innate immune mechanisms, especially the NOD-like receptor family, remain poorly characterized in this species. Therefore, understanding the function of key immune genes such as the *NOD1* gene in snakehead is essential for developing effective disease control strategies and supporting sustainable aquaculture. In the present study, the full-length *NOD1* gene in snakehead was characterized, which possesses conserved CARD, NACHT, and LRR domains and exhibits close homology and conserved genomic synteny with Perciformes. The expression analysis revealed that *NOD1* was highly expressed in the intestine and spleen, while *N. seriolae* challenge induced significant upregulation in the gill and spleen. Subcellular localization confirmed that *NOD1* was mainly distributed in the cytoplasm. Functional verification demonstrated that *NOD1* activated NF-κB in a dose- and time-dependent manner, and specifically responded to iE-DAP rather than other ligands. Furthermore, the complete structural domains of *NOD1* were indispensable for maintaining its NF-κB-activating capacity, and only the full-length *NOD1* exerted effective activation under iE-DAP stimulation. In addition, *NOD1* collaborated with the downstream adaptor *RIPK2* to synergistically enhance NF-κB activity, and direct protein interaction existed between *NOD1* and *RIPK2*, though their binding affinity was not enhanced by iE-DAP or PGN stimulation.

## 2. Materials and Methods

### 2.1. Fish and Cell Lines

The snakeheads were purchased from Shandong Province, China, and weighed approximately 20 g each. Initially, the fish were acclimated in a recirculating freshwater system for 7 days prior to the bacterial infection experiments. During the acclimation period, the water quality parameters were maintained as follows: pH 7.8 ± 0.5, temperature 22 ± 0.5 °C, salinity 0 ppt, and dissolved oxygen 7.0 ± 0.5 mg/L. The snakeheads used in this study were purchased from a certified breeding farm, where routine pathogen and disease surveillance is conducted to ensure the health status of the stock. No obvious signs of disease were observed in the snakeheads during the acclimatization period; furthermore, no signs of disease were observed in the control group during the experiment. Nine organs/tissues including blood, brain, skin, liver, muscle, head kidney, gill, spleen and intestine were collected from four healthy snakeheads for constitutive expression analysis. Each tissue from each fish was divided into three equal portions, respectively. Thus, three independent pooled samples were obtained per tissue, each containing tissue portions from all four fish. For the in vivo stimulation experiments, liver, spleen, head kidney, skin, gill, and intestine samples were collected at 6, 12, 24, 48 and 72 h post-infection. *N. seriolae* was administered via intraperitoneal (IP) injection at a dose of 100 μL per fish (1 × 10^8^ CFU/mL, i.e., 1 × 10^7^ CFU per fish). The control fish received an equal volume of PBS. For the in vivo challenge experiments, 15 fish were sampled at each time point, for a total of 90 fish. During the 72 h post-infection period, all the fish were starved to avoid any effect of feeding on immune parameters.

Epithelioma papulosum cyprini (EPC) cells were maintained in M199 medium (Hyclone, Logan, UT, USA) containing 10% fetal bovine serum (FBS, HyClone, Logan, UT, USA) at 28 °C with 5% CO_2_. HEK 293T cells (human embryonic kidney 293T) were grown in DMEM (HyClone, Logan, UT, USA) with 10% FBS at 37 °C in a 5% CO_2_ environment. *N. seriolae* was inoculated in Ogawa medium at 28 °C with 180 rpm.

All the experiments were performed in accordance with local government regulations, and all procedures involving fish were conducted following the ethical guidelines established by the Animal Ethics Committee of Qingdao Agricultural University (QAU-IACUC-2023-021).

### 2.2. RNA Extraction, cDNA Synthesis and Quantitative Real-Time PCR

Total RNA was isolated using RNAiso reagent (Takara, Shiga-ken, Japan), and the first-strand cDNA template was synthesized using a PrimeScript™ 1st strand cDNA Synthesis Kit (Takara, Shiga-ken, Japan) according to the manufacturer’s instructions. Quantitative real-time PCR (qRT-PCR) was performed on a QuantStudiO™ 5 real-time PCR instrument (Thermo Fisher Scientific, Asheville, NC, USA) using the following cycling conditions: initial incubation at 95 °C for 2 min, followed by 40 cycles of 95 °C for 5 s, 60 °C for 30 s, and 65 °C for 5 s. The qPCR reaction mixture consisted of 5 μL of 2 × SYBR green pro taq HS premix (Accurate, Changsha, China), 1 μL of cDNA template, 0.4 μL of forward primer (10 μM), 0.4 μL of reverse primer (10 μM), and 3.2 μL of nuclease-free water. The specific primers are listed in Table 1. The relative expression levels were calculated using the 2^−ΔΔCt^ method and normalized to β-actin gene.

### 2.3. Bioinformatic Analyses and Gene Cloning

The gene and protein sequences used in bioinformatics were compared with homologous sequences through the Basic Local Alignment Search Tool (BLAST) version 2.17.0 on the website of the National Center for Biotechnology Information (NCBI, http://blast.ncbi.nlm.nih.gov/Blast.cgi (accessed on 5 December 2024)). Multiple alignments of proteins were performed using Clustal W (version 2.1). A phylogenetic tree was constructed using the neighbor-joining (NJ) method in MEGA X software (version 10.2.4), with 1000 times bootstrap.

Each PCR mixture contained 12.5 μL of PrimerSTAR (Takara, Shiga-ken, Japan), 1.0 μL of cDNA, 1.0 μL of forward primer, 1.0 μL of reverse primer, and nuclease-free water to bring the final volume to 25 μL. Following amplification, the products were visualized via agarose gel electrophoresis, then purified with the FastPure^®^ Gel DNA Extraction Mini Kit (Vazyme, Nanjing, China). Subsequently, they were ligated into the pMD18-T vector (Takara, Shiga-ken, Japan) and introduced into competent DH5α. Positive transformants were sequenced by Beijing Tsingke Biotech Co., Ltd. (Beijing, China), and the primer sequences are provided in Table 1.

### 2.4. Subcellular Localization

The ORF of *NOD1* and *RIPK2* genes were subcloned into the pEGFP-N2 vector to generate *NOD1*-GFP and *RIPK2*-GFP fluorescent fusion expression vectors. HEK 293T cells were plated into 24-well plates and incubated overnight. *NOD1*-GFP and *RIPK2*-GFP expression plasmids (1 μg/well) were transfected into the HEK 293T cells using Lipofectamine 2000 (Invitrogen, Carlsbad, CA, USA) according to the manufacturer’s protocol. At 24 h post-transfection (hpt), the cells were fixed with 4% paraformaldehyde for 10 min, and permeabilized with 1% Triton X-100 solution at room temperature for 10 min. DAPI was added to the coverslip for observation under a confocal laser scanning microscope (ZEISS, Jena, Germany).

### 2.5. Co-Immunoprecipitation (Co-IP) and Western Blotting

The ORFs of *NOD1* and *RIPK2* were inserted into the pcDNA3.1-myc-His(A) and p3×FLAG-CMV14 vectors with Myc and Flag tags, respectively. *NOD1*-Myc and *RIPK2*-Flag were transfected into HEK 293T cells by using Lipofectamine 2000. The cells were collected at 48 hpt and lysed 15 min at 4 °C in RIPA lysis buffer (Beyotime, Shanghai, China) supplemented with PMSF (Beyotime, Shanghai, China). After centrifugation, 40 μL mixed liquid was taken as the input sample. Anti-Myc or anti-Flag immunomagnetic beads (Beyotime, Shanghai, China) were added to the remaining samples and incubated overnight at 4 °C for Co-IP. Upon completion, the samples received 1× SDS loading buffer and were then boiled for 10 min. Subsequent separation of protein samples occurred via 8% or 12% SDS-PAGE gels, followed by transfer onto a 0.22 μm polyvinylidene fluoride membrane (Millipore, Jaffrey, NH, USA). The membrane, after being rinsed with PBST buffer, was blocked for 1 h at room temperature using 5% non-fat milk, then incubated overnight with primary antibody at a 1:5000 dilution. Following another PBST wash, the membrane was exposed to secondary antibody for 1 h. Lastly, visualization and detection were carried out with the ChemiDoc™ MP system (Bio-Rad, Irvine, CA, USA).

### 2.6. Luciferase Activity Assays

The luciferase reporter vectors of NF-κB, pRL-TK, and eukaryotic expression mutant plasmids of NOD, including NOD-ΔCARD, NOD-ΔLRRs, NOD-CARD, NOD-ΔNACHT, and NOD-LRRs, were constructed. A series of plasmid mixtures were transfected into EPC cells seeded in 24-well plates via Lipofectamine 2000 transfection reagent. The empty vector was employed as the control group, and the cells were stimulated with LPS (10 μg/mL), LTA (10 μg/mL), poly(I:C) (10 μg/mL), PGN (10 μg/mL), and iE-DAP (10 μg/mL) (stock concentration: 5 mg/mL for all ligands). LPS, LTA and PGN were purchased from Sigma-Aldrich (St. Louis, MO, USA), while poly(I:C) and iE-DAP were purchased from Invitrogen (Carlsbad, CA, USA). The cells in each well were collected at 24 hpt and washed once with PBS. Subsequently, 200 μL of Lysis Buffer (Beyotime, Shanghai, China) was added to lyse the cells. The luciferase activity was measured using the dual-luciferase reporter gene assay kit (Beyotime, Shanghai, China) with a microplate reader.

### 2.7. Statistical Analyses

Statistical analysis of the experimental data was performed with SPSS 20.0 software. Prior to statistical analysis, all data were checked for normality using the Shapiro–Wilk test and for homogeneity of variances using Levene’s test. The results met the assumptions for parametric tests. Student’s *t*-test was then employed for comparisons between two groups, and one-way ANOVA followed by Duncan’s multiple range test was used for multiple comparisons. Each experiment was conducted a minimum of three times, and the results are expressed as means ± standard error (SE). Differences reaching statistical significance are denoted by * *p* < 0.05.

## 3. Results

### 3.1. Identification of NOD1 in Snakehead

**Figure 1:**
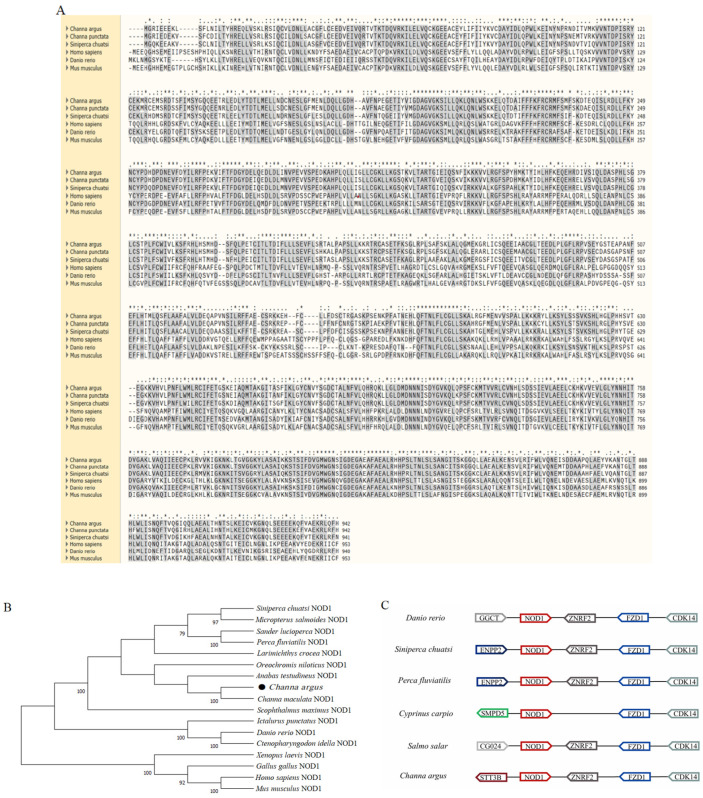
Identification of *NOD1* in snakehead. (**A**) A multi-sequence comparison of snakehead *NOD1* and other species. Asterisks (*) indicate identical residues; dots (.) indicate conservative substitutions. (**B**) Phylogenetic analysis of *NOD1* in snakehead and other vertebrates. Phylogenetic analysis was done using MEGA X by performing the neighbor-joining method with 1000 bootstrap replicates. GenBank accession numbers of selected *NOD1* amino acid sequences: *Homo sapiens NOD1* (AAD28350.1), *Mus musculus NOD1* (AAN52479.1), *Gallus gallus NOD1* (AFS49704.1), *Xenopus laevis NOD1* (XP_018124808.1), *Danio rerio NOD1* (XP_002665106.3), *Siniperca chuatsi NOD1* (XP_044063118.1), *Micropterus salmoides NOD1* (XP_038568219.1), *Sander lucioperca NOD1* (XP_035862690.1), *Perca fluviatilis NOD1* (XP_039661787.1), *Larimichthys crocea NOD1* (XP_010737413.3), *Oreochromis niloticus NOD1* (XP_003446247.1), *Anabas testudineus NOD1* (XP_026229064.1), *Channa punctata NOD1* (QDH76353.1), *Scophthalmus maximus NOD1* (XP_035485565.2), *Ictalurus punctatus NOD1* (NP_001186996.1), *Ctenopharyngodon idella NOD1* (XP_051720971.1). (**C**) Synteny analysis of *NOD1* in snakehead.

The *NOD1* gene identified in snakehead contains an open reading frame of 2829 bp, encoding 943 amino acids. Multiple sequence alignment results showed that the snakehead *NOD1* protein was highly similar to that of other teleosts. (Figure 1A). Phylogenetic analysis revealed that snakehead *NOD1* protein shares high sequence similarity with *NOD1* from other vertebrates, exhibiting particularly close homology with orthologs from Perciformes (Figure 1B). Moreover, the *NOD1* locus is evolutionarily conserved and arranged in tandem with the *ZNRF2* gene (Figure 1C).

### 3.2. Expression of the NOD1 Gene

**Figure 2:**
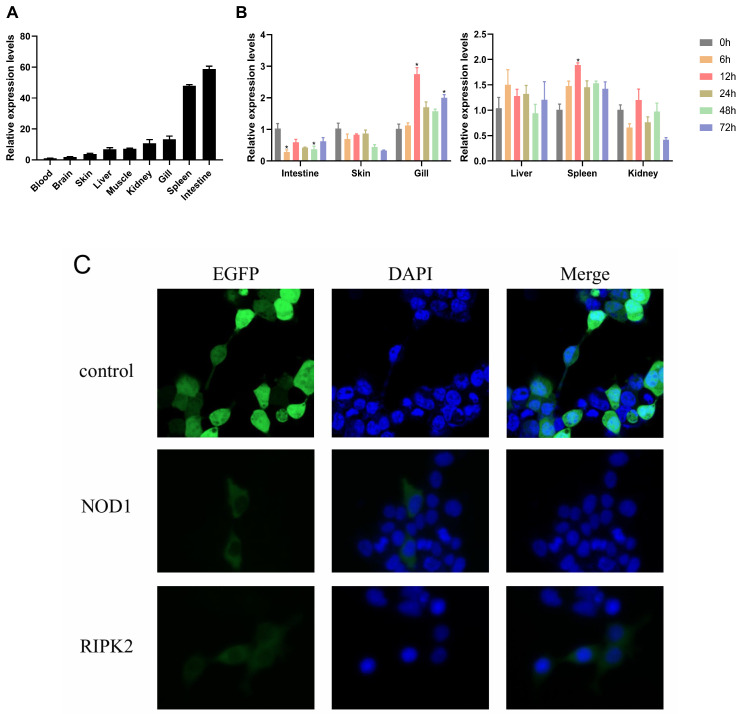
The expression level of *NOD1* in different tissues. (**A**) Total RNA was extracted from nine tissues (brain, gill, muscle, skin, intestine, blood, liver, spleen and kidney) and analyzed by qPCR to examine the tissue expression of *NOD1*. The relative mRNA level in each sample was normalized to the level of β-actin and the expression levels were calibrated against tissue that had the lowest expression level. (**B**) The expression level of *NOD1* after *N. seriolae* infection. The results were expressed as the means ± standard error from three independent triplicated experiments and significant difference was indicated with * *p* < 0.05. (**C**) The subcellular localization of *NOD1* and *RIPK2* in the HEK293 cells. The cells were transfected with pEGFP-N2, *NOD1*-GFP and *RIPK2*-GFP. After 24 h, the cells were fixed and the nucleus stained with DAPI. Green represents the target gene; blue represents the nucleus.

To explore the expression pattern of *NOD1* in snakehead, qRT-PCR was performed to detect the transcription levels of *NOD1*. The results showed that *NOD1* was expressed in the liver, spleen, head kidney, intestine, skin, gill, muscle, blood, and brain with variable expression levels. Notably, low expression levels were detected in the blood and brain, while high expression was observed in the intestine (58.8-fold), followed by the spleen (47.9-fold) (Figure 2A).

Following intraperitoneal injection of *N. seriolae*, liver, spleen, head kidney, intestine, skin, and gill samples were collected and the expression of *NOD1* was analyzed by qRT-PCR. Upon *N. seriolae* infection, the transcription of *NOD1* was significantly upregulated in the gill and spleen, whereas its expression was downregulated in the intestine, skin, and head kidney, but remained unchanged in the liver (Figure 2B).

To determine the subcellular localization of snakehead *NOD1* protein, the ORF of *NOD1* was cloned into the pEGFP-N2 vector with GFP tag. The recombinant plasmid *NOD1*-N2 and the empty pEGFP-N2 control plasmid were separately transfected into HEK 293T cells. At 24 hpt, fluorescence microscopic observation revealed that the GFP signal of the empty vector was uniformly distributed throughout the cytoplasm and nucleus. In contrast, the *NOD1*-GFP fusion protein was predominantly localized in the cytosolic (Figure 2C).

### 3.3. The Activation of NF-κB by NOD1

**Figure 3:**
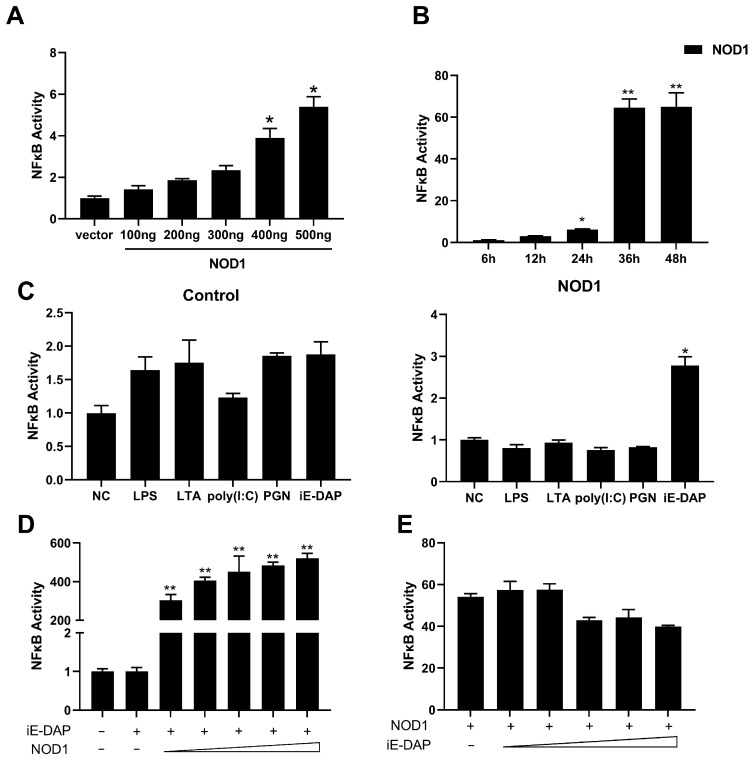
The activation of NF-κB by *NOD1*. EPC cells were co-transfected with 100 ng, 200 ng, 300 ng, 400 ng and 500 ng *NOD1* expression plasmids respectively along with NF-κB reporter gene plasmids to perform the concentration gradient experiment (**A**), and co-transfected with *NOD1* expression plasmids and NF-κB reporter gene plasmids into cells to perform the time gradient experiment (**B**); the luciferase activity of NF-κB was detected at 24 h post-transfection. (**C**) EPC cells were transfected with *NOD1* expression plasmids or empty control vectors together with NF-κB and pRL-TK, and then transfected with LTA, poly(I:C), iE-DAP and PGN to stimulate the cells; at 24 h post-transfection, the luciferase activity of NF-κB was detected. (**D**) After co-transfecting cells with *NOD1* plasmids of varying concentrations, NF-κB, and pRL-TK for 24 h, the cells were stimulated with iE-DAP, and luciferase activity was measured 6 h after stimulation. (**E**) Cells were co-transfected with *NOD1* expression plasmids, NF-κB and pRL-TK internal reference plasmid 24 h later, and then different concentrations of iE-DAP stimulated the cells intracellularly; the luciferase activity of NF-κB was detected at 24 h post-stimulation. The data are expressed as mean ± SE from three independent experiments, with each sample measured in triplicate and significant difference indicated with * *p* < 0.05 and ** *p* < 0.01.

To investigate the PAMPs specifically recognized by snakehead *NOD1* during antimicrobial immune responses, the *NOD1* expression plasmid or empty control plasmid was co-transfected with the NF-κB luciferase reporter and the internal control plasmid pRL-TK into the EPC cells. At 24 hpt, NF-κB activity was examined following treatment with different stimuli, including poly(I:C), LPS, iE-DAP, PGN, and LTA, at various concentrations and time points. The results showed that NF-κB activity increased in a dose-dependent manner with the escalating dosage of the *NOD1* expression plasmid (Figure 3A), and significant activation of NF-κB was observed at 48 h after transfection (Figure 3B). Compared with the control groups, only iE-DAP was capable of triggering *NOD1*-mediated NF-κB activation (Figure 3C), and this activating effect was further enhanced with the increase in *NOD1* plasmid dosage (Figure 3D); the other ligands failed to induce notable NF-κB activation (Figure 3C). Furthermore, variations in iE-DAP concentration exerted no significant influence on *NOD1*-dependent NF-κB activation (Figure 3E).

**Figure 4:**
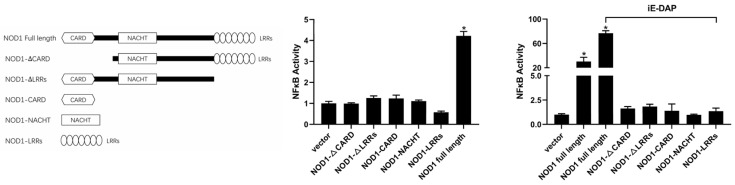
A schematic representation of full-length *NOD1* and the variants used in this study. NF-κB promoter activation by *NOD1* and its variants. The data are expressed as mean ± SE from three independent experiments, with each sample measured in triplicate, and * indicating *p* < 0.05.

To further investigate the functional roles of distinct domains of snakehead *NOD1* protein in regulating NF-κB activation, a series of eukaryotic expression plasmids carrying truncated *NOD1* mutants were constructed (Figure 4). Dual-luciferase reporter assays demonstrated that any domain deletion completely abolished the ability of *NOD1* to activate NF-κB. Moreover, individual overexpression of the NACHT domain or LRR domains failed to efficiently induce NF-κB activation (Figure 4). Upon stimulation with iE-DAP, significant NF-κB activation was only detected in the cells transfected with the full-length *NOD1* (Figure 4).

### 3.4. Interaction of NOD1 and RIPK2

**Figure 5:**
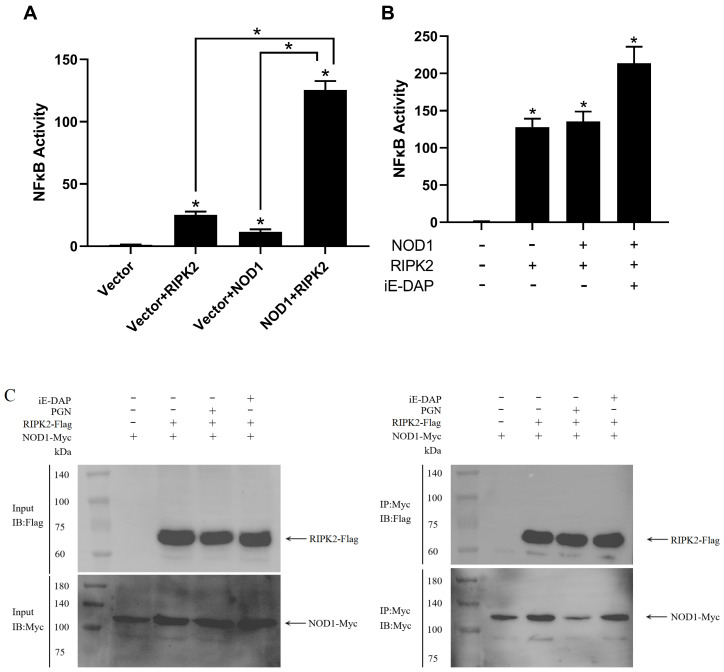
The association between *NOD1* and *RIPK2*. (**A**) The association between *NOD1* and *RIPK2* in NF-κB activation. (**B**) *RIPK2* and *NOD1* plasmids were transfected along with the NF-κB plasmid into the cells, then the cells were stimulated with iE-DAP, and 6 h later, the luciferase activity of NF-κB was detected. pRL-TK as the internal control, and the data are expressed as mean ± SE of three independent experiments with each sample measured in triplicate. * indicates *p* < 0.05. (**C**) The interaction of *RIPK2* with *NOD1* through co-immunoprecipitation (Co-IP) analysis.

In mammals, *RIPK2* has been well characterized as the key adaptor protein downstream of the *NOD1*-mediated signaling pathway. Accordingly, the correlation between snakehead *RIPK2* and *NOD1* signaling cascade was investigated in the present study. Dual-luciferase reporter assays showed that individual overexpression of either *RIPK2* or *NOD1* triggered mild activation of NF-κB. Notably, co-transfection of *RIPK2* and *NOD1* expression plasmids resulted in a significant enhancement of NF-κB activation and NF-κB activity increased dramatically following iE-DAP stimulation (Figure 5A,B). Subsequently, *RIPK2* and *NOD1* were co-transfected into HEK293T cells, and their protein interaction was determined via co-immunoprecipitation (Co-IP). The results verified a direct binding interaction between *NOD1* and *RIPK2*. Moreover, this interaction affinity was not further strengthened upon stimulation with iE-DAP or PGN (Figure 5C). The original, unmodified scans of the Western blot membranes are provided in Supplementary Materials.

## 4. Discussion

As mentioned earlier, the innate immune system serves as the body’s first line of defense against invading pathogenic microorganisms and plays a pivotal role in the recognition of these microorganisms and the immune response during the early stages of infection [33,34]. NLRs are a class of PRRs that are primarily localized in the cytoplasm of host cells and are relatively conservative in evolutionary terms; homologous genes have been identified in both fish and mammals [35,36,37]. In mammals, *NOD1* effectively recognizes iE-DAP—a PGN motif found in Gram-negative bacteria and certain Gram-positive bacteria—within the PGN structure of pathogens that have entered host cells, thereby activating downstream NF-κB signaling pathways [10,38,39]. However, there is currently limited research on the role of *NOD1* in fish, and we do not yet fully understand its specific functions in ligand recognition specificity, adaptor protein usage, and tissue expression profiles, as well as its downstream signaling pathways. Hence, the main goal of the present research is to systematically detect and characterize the expression profiles of snakehead *NOD1*, and to investigate its role in inflammatory responses. Synteny analysis suggests that *NOD1* is evolutionarily conserved and may play a critical role in host defense throughout evolution. The preservation of genetic data implies that snakehead *NOD1* could exhibit comparable functionality to its mammalian counterpart, with the ability to detect conserved bacterial peptidoglycan motifs and initiate inflammatory pathways.

Sequence analysis revealed that *NOD1* lacks a signal peptide, indicating that it is an intracellular protein; this finding was further validated by subcellular localization experiments. This is consistent with findings from studies in higher vertebrates indicating that *NOD1* is an intracellular pattern recognition receptor [40]. In mammals, the *NOD1* protein primarily consists of three distinct functional domains: CARD, NACHT, and LRR. Among these, the CARD is responsible for binding downstream adaptor proteins, while the LRR domain is responsible for recognizing and binding antigens [41]. Similar to the mammalian *NOD1* protein, the *NOD1* protein in snakehead consists of the typical CARD-NACHT-LRR structure, and phylogenetic analysis also indicates a close evolutionary relationship with mammalian *NOD1* proteins.

Studies in other teleosts have shown that the *NOD1* gene was expressed in all examined tissues, and the *NOD1* protein was highly enriched in immune-related tissues, suggesting that *NOD1* played an important role in the host immune response [17,42,43,44,45,46]. Consistent with previous studies, *NOD1* was expressed in all nine tissues of snakehead, with the highest expression levels in the intestine, followed by the spleen, gill, and head kidney. Similar to this, zebrafish *NOD1* was highly expressed in the intestine, spleen, and liver [37]; in Chinese perch, *NOD1* enrichment was detected in the head kidney, gill, and spleen [47], and *NOD1* was most highly expressed in the liver, gill, and skin of miiuy croaker (*Miichthys miiuy*) [48]. These are the primary mucosal immune tissues and classical immune organs of fish, suggesting that *NOD1* may play an indispensable role in the innate immune system of the snakehead [49]. To further understand the cellular basis of this tissue distribution pattern, we compared our findings with known *NOD1*-expressing cell types in higher vertebrates. Our qPCR analysis revealed high *NOD1* transcript abundance in the intestine, spleen, and gill of snakehead. While single-cell resolution data are not yet available for snakehead, comparative insights can be drawn from single-cell RNA sequencing and well-established expression studies in higher vertebrates. In humans, *NOD1* is expressed in a broad range of cell types, including intestinal epithelial cells [50], as well as immune cells such as alveolar macrophages, monocyte-derived macrophages, and peripheral blood monocytes [51]. *NOD1* expression has also been detected in CD14^+^ monocytes, CD1a^+^ immature dendritic cells [52], and B lymphocytes [53]. Accordingly, the abundant *NOD1* transcripts we observed in the snakehead spleen and gill likely reflect expression in analogous cell populations, particularly macrophages and lymphocytes, which are central to antibacterial immune surveillance. The highest expression in the intestine is likely attributed to its role as a major mucosal immune barrier, continuously exposed to commensal microbiota and dietary antigens, thus requiring heightened immune surveillance [50]. Although single-cell transcriptomic studies in teleost fish (e.g., turbot [54], largemouth bass [55], and large yellow croaker [56]) have successfully resolved immune cell landscapes, none of these studies have yet directly assigned *NOD1* expression to specific cell types at single-cell resolution. Therefore, while the overall tissue expression pattern of snakehead *NOD1* is broadly consistent with the cellular distribution profile known for mammalian *NOD1*, future studies employing in situ hybridization, single-cell RNA sequencing, or immunohistochemistry will be necessary to identify the precise *NOD1*-expressing cell populations in snakehead tissues.

Earlier investigations demonstrated that *NOD1* serves a critical function in host innate defense against bacterial pathogens. For example, inhibiting *NOD1* expression in zebrafish impairs the ability of juvenile fish to resist bacterial proliferation [57]; in orange-spotted grouper (*Epinephelus coioides*), *NOD1* expression in the spleen significantly increased 72 h after infection with *Vibrio alginolyticus* as measured by qRT-PCR [46]. After infection with *Vibrio anguillarum*, *NOD1* expression in the spleen of miiuy croaker exhibited a trend of initially decreasing followed by an increase, also determined by qRT-PCR [48]. In this study, we further explored the expression changes of *NOD1* after bacterial infection and found that the expression of *NOD1* was upregulated at certain time points in the spleen, liver, and gill tissues after *N. seriolae* infection, indicating that *NOD1* is actively involved in antibacterial immune responses. In contrast, *NOD1* expression was significantly reduced in the intestine. Several factors may account for this downregulation: first, *N. seriolae* infection can cause intestinal epithelial cell necrosis and tissue damage, directly reducing the number of *NOD1*-expressing cells [58]; second, as an intracellular pathogen, *N. seriolae* may induce apoptosis of macrophages, which are major *NOD1*-expressing cells, thereby diminishing the expression level [59]; third, the pathogen possesses immunosuppressive and immune evasion capabilities, potentially leading to transient suppression of immune gene expression in mucosal tissues [60]. Pathogen infection may induce apoptosis or trigger the early stages of programmed cell death, leading to a decline in overall protein synthesis capacity, which in turn affects the expression of *NOD1* as a specific protein. Since the present study largely relies on in vitro overexpression models, further in vivo validation using gene knockdown or knockout approaches is needed to confirm the physiological role of *NOD1*.

*NOD1* primarily recognizes antigens through its C-terminal LRR domain and further interacts with the adaptor protein *RIPK2* via its N-terminal CARD to activate downstream signaling pathways [61]. Our study revealed that snakehead *NOD1* significantly activates the downstream NF-κB signaling pathway, and that the induced NF-κB activity increases in a concentration-dependent manner with increasing *NOD1* levels. This finding is consistent with observations reported in other teleost fish [62,63,64]. Furthermore, iE-DAP stimulation resulted in significantly higher NF-κB activity compared to the other four ligands or the control group, and a marked enhancement of NF-κB signaling pathway activation was observed upon co-exposure to *NOD1* and iE-DAP. Similar results were also observed in golden pompano (*Trachinotus ovatus*) and Nile tilapia (*Oreochromis niloticus*) [62,63].

## 5. Conclusions

Taken together, the snakehead *NOD1* gene was obtained by cloning in this study. Through sequence analysis, it was found that *NOD1* encodes 943 amino acids. Phylogenetic analysis showed that the snakehead *NOD1* protein was closely related to the Perciformes. At the same time, the qPCR results indicated that *NOD1* was expressed in all tissues. In the case of bacterial infection, *NOD1* expression was upregulated in the spleen and gill tissues. The results from the dual-luciferase reporter assays showed that overexpression of *NOD1* significantly induced NF-κB activity and activated NF-κB in a dose- and time-dependent manner. Moreover, *NOD1* synergistically enhanced NF-κB activity with the downstream adaptor protein *RIPK2*. These findings suggest that snakehead *NOD1* contributes to antibacterial innate immune signaling through NF-κB activation.

## Figures and Tables

**Table 1:** The primers used in this study.

Primer	Sequence (5′ to 3′)
*NOD1*-F	ATGGGTCGAATAGAAGAGGAG
*NOD1*-R	TCAGTGGAACTGCAGTCTC
Myc-*NOD1*-F	TCCAGTGTGGTGGAATTCATGGGTCGAATAGAAGAGGAG
Myc-*NOD1*-R	GGGCCCTCTAGACTCGAGGTGGAACTGCAGTCTC
Myc-*NOD1*-ΔCARD-F	TCCAGTGTGGTGGAATTCATGTGGCTGAAAGAAATCAACTACA
Myc-*NOD1*-△LRR-R	GGGCCCTCTAGACTCGAGATGCTGTAGCACAAAGTTCAGA
Myc-*NOD1*-CARD-R	GGGCCCTCTAGACTCGAGTTCTTTCAGCCATGGCTGGA
Myc-*NOD1*-NACHT-F	TCCAGTGTGGTGGAATTCATGGAAGGTGAAACCATTTATGTG
Myc-*NOD1*-NACHT-R	GGGCCCTCTAGACTCGAGGTGCTCCTGCTCCTTAAAGT
Myc-*NOD1*-LRRs-F	TCCAGTGTGGTGGAATTCATGCGTCAGAAGCTCCTGGGGC
*NOD1*-GFP-F	CGTCAGATCCGCTAGCATGGGTCGAATAGAAGAGGAG
*NOD1*-GFP-R	ACGGCCGGTGGATCCGGTGGAACTGCAGTCTC
*RIPK2*-F	ATGGAGCCTGCGGCTATGGGCTG
*RIPK2*-R	CTACATATTCCTGGGGATATT
Flag-*RIPK2*-F	ACCGTCAGAATTAAGCTTATGGAGCCTGCGGCTATGGGCTG
Flag-*RIPK2*-R	GTAGTCAGCCCGGGATCCCATATTCCTGGGGATATT
*RIPK2*-GFP-F	CGTCAGATCCGCTAGCATGGAGCCTGCGGCTATGGGCTG
*RIPK2*-GFP-R	ACGGCCGGTGGATCCGCATATTCCTGGGGATATT
q*NOD1*-F	GTCAGACAGCAGCATTGAGG
q*NOD1*-R	CCAATCTTGACGACTCGCAG
q*RIPK2*-F	GAAGCTGACCGACCTGTACT
q*RIPK2*-R	TGCAGAAGAACTCAGGCTCA
β-actin-F	CACTGTGCCCATCTACGAG
β-actin-R	CCATCTCCTGCTCGAAGTC

## Data Availability

The original contributions presented in this study are included in the article. Further enquiries can be directed to the corresponding author.

## Supplementary Materials

The following supporting information can be downloaded at: https://www.mdpi.com/article/10.3390/biology15120942/s1.

## References

[1] Chen R., Zou J., Chen J., Zhong X., Kang R., Tang D. (2025). Pattern Recognition Receptors: Function, Regulation and Therapeutic Potential. Signal Transduct. Target. Ther..

[2] Philpott D.J., Sorbara M.T., Robertson S.J., Croitoru K., Girardin S.E. (2014). Nod Proteins: Regulators of Inflammation in Health and Disease. Nat. Rev. Immunol..

[3] Tsankov B.K., Luchak A., Carr C., Philpott D.J. (2024). The Effects of Nod-Like Receptors on Adaptive Immune Responses. Biomed. J..

[4] Chou W.-C., Jha S., Linhoff M.W., Ting J.P.-Y. (2023). The Nlr Gene Family: From Discovery to Present Day. Nat. Rev. Immunol..

[5] Gong T., Liu L., Jiang W., Zhou R. (2020). Damp-Sensing Receptors in Sterile Inflammation and Inflammatory Diseases. Nat. Rev. Immunol..

[6] Kanneganti T.-D., Özören N., Body-Malapel M., Amer A., Park J.-H., Franchi L., Whitfield J., Barchet W., Colonna M., Vandenabeele P. (2006). Bacterial Rna and Small Antiviral Compounds Activate Caspase-1 through Cryopyrin/Nalp3. Nature.

[7] Mariathasan S., Newton K., Monack D.M., Vucic D., French D.M., Lee W.P., Roose-Girma M., Erickson S., Dixit V.M. (2004). Differential Activation of the Inflammasome by Caspase-1 Adaptors Asc and Ipaf. Nature.

[8] Meunier E., Broz P. (2017). Evolutionary Convergence and Divergence in Nlr Function and Structure. Trends Immunol..

[9] Sundaram B., Tweedell R.E., Kumar S.P., Kanneganti T.-D. (2024). The Nlr Family of Innate Immune and Cell Death Sensors. Immunity.

[10] Caruso R., Warner N., Inohara N., Núñez G. (2014). *NOD1* and Nod2: Signaling, Host Defense, and Inflammatory Disease. Immunity.

[11] Inohara N., Koseki T., Lin J., del Peso L., Lucas P.C., Chen F.F., Ogura Y., Núñez G. (2000). An Induced Proximity Model for Nf-Kappa B Activation in the *NOD1*/Rick and Rip Signaling Pathways. J. Biol. Chem..

[12] Sahoo B.R. (2020). Structure of Fish Toll-Like Receptors (Tlr) and Nod-Like Receptors (Nlr). Int. J. Biol. Macromol..

[13] Swain B., Miryala K.R. (2025). NOD-like Receptors in Fish: Evolution, Structure, Immune signaling, and Targeting for Aquaculture Vaccine Adjuvants. Front. Immunol..

[14] Wu X.M., Chen W.Q., Hu Y.W., Cao L., Nie P., Chang M.X. (2018). Rip2 Is a Critical Regulator for Nlrs Signaling and Mhc Antigen Presentation but Not for Mapk and Pi3k/Akt Pathways. Front. Immunol..

[15] Wu X.M., Zhang J., Li P.W., Hu Y.W., Cao L., Ouyang S., Bi Y.H., Nie P., Chang M.X. (2020). *NOD1* Promotes Antiviral Signaling by Binding Viral Rna and Regulating the Interaction of Mda5 and Mavs. J. Immunol..

[16] Xie J., Belosevic M. (2015). Functional Characterization of Receptor-Interacting Serine/Threonine Kinase 2 (Rip2) of the Goldfish (*Carassius auratus* L.). Dev. Comp. Immunol..

[17] Xie J., Hodgkinson J.W., Katzenback B.A., Kovacevic N., Belosevic M. (2013). Characterization of Three Nod-Like Receptors and Their Role in Antimicrobial Responses of Goldfish (*Carassius auratus* L.) Macrophages to Aeromonas Salmonicida and Mycobacterium Marinum. Dev. Comp. Immunol..

[18] Jang J.H., Kim H., Kim Y.J., Cho J.H. (2016). Molecular Cloning and Functional Analysis of Nucleotide-Binding Oligomerization Domain-Containing Protein 1 in Rainbow Trout, *Oncorhynchus mykiss*. Fish Shellfish Immunol..

[19] Chen D., Zhu H., Lu L., Chen Y., Zhang X., Huang X., Ouyang P., Geng Y., Li Z. (2024). Identification, Characterization and the Inflammatory Regulating Effect of *NOD1*/2 in Sturgeon. Fish Shellfish Immunol..

[20] Peng X.Y., Wang K.L., Li L., Li B., Wu X.Y., Zhang Z.W., Li N., Liu L.H., Nie P., Chen S.N. (2024). Transcription of *NOD1* and Nod2 and Their Interaction with Card9 and *RIPK2* in Ifn Signaling in a Perciform Fish, the Chinese Perch, *Siniperca chuatsi*. Front. Immunol..

[21] Teng J., Zhao Y., Meng Q.L., Zhu S.R., Chen H.J., Xue L.Y., Ji X.S. (2022). Transcriptome analysis in the spleen of Northern Snakehead (*Channa argus*) challenged with Nocardia seriolae. Genomics.

[22] Zhang W., Zhou K., Huang L., Yang N., Lin L., Chen L., Yao J., Dong M., Shen J., Pan X. (2024). Biological characteristics and pathogenicity comparison of Nocardia seriolae isolated from *Micropterus salmoides* and *Channa argus*. Front. Vet. Sci..

[23] Wang G.L., Xu Y.J., Jin S., Zhu J.L., Zhu W.Y. (2009). Research on the Nocardiosis and Pathogen in Reared Snakehead, *Ophiocephalus argus* Cantor. Acta Hydrobiol. Sin..

[24] Zhang N., Zhang H., Dong Z., Wang W. (2022). Molecular Identification of Nocardia seriolae and Comparative Analysis of Spleen Transcriptomes of Hybrid Snakehead (*Channa maculata* Female × *Channa argus* Male) with Nocardiosis Disease. Front. Immunol..

[25] Zhou T., Cai P., Li J., Dan X., Li Z. (2024). Pathological Variations and Immune Response in *Channa argus* Infected with Pathogenic Nocardia seriolae strain. Fish Shellfish. Immunol..

[26] Liu Y., Chen G., Xia L., Lu Y. (2023). A Review on the Pathogenic Bacterium *Nocardia seriolae*: Aetiology, Pathogenesis, Diagnosis And Vaccine Development. Rev. Aquac..

[27] Lechevalier M.P., Horan A.C., Lechevalier H. (1971). Lipid Composition in the Classification of Nocardiae and Mycobacteria. J. Bacteriol..

[28] Lechevalier M.P., Lechevalier H. (1970). Chemical composition as a criterion in the classification of aerobic actinomycetes. Int. J. Syst. Evol. Microbiol..

[29] Zhou Z.-Y., Bai S.-J., He J., Xiong Q.-X., Zhong Z.-D., Lu C.-W., Kuang L.-F., Jian Z.-R., Gu J.-L., Liu M.-Z. (2025). Pathogenicity, ultrastructure and genomics analysis of *Nocardia seriolae* isolated from largemouth bass (*Micropterus salmoides*). Microb. Pathog..

[30] Chamaillard M., Hashimoto M., Horie Y., Masumoto J., Qiu S., Saab L., Ogura Y., Kawasaki A., Fukase K., Kusumoto S. (2003). An Essential Role for *NOD1* in Host Recognition of Bacterial Peptidoglycan Containing Diaminopimelic Acid. Nat. Immunol..

[31] Girardin S.E., Boneca I.G., Carneiro L.A.M., Antignac A., Jéhanno M., Viala J., Tedin K., Taha M.-K., Labigne A., Zäthringer U. (2003). *NOD1* Detects a Unique Muropeptide From Gram-Negative Bacterial Peptidoglycan. Science.

[32] Han H., Kwak M., Ha S., Yang S., Kim J.D., Cho K., Kim T., Cho M.Y., Kim B., Jung S. (2019). Genomic Characterization of *Nocardia seriolae* Strains Isolated from Diseased Fish. Microbiologyopen.

[33] Magnadóttir B. (2006). Innate Immunity of Fish (Overview). Fish Shellfish Immunol..

[34] Dezfuli B.S., Lorenzoni M., Carosi A., Giari L., Bosi G. (2023). Teleost Innate Immunity, an Intricate Game between Immune Cells and Parasites of Fish Organs: Who Wins, Who Loses. Front. Immunol..

[35] Meylan E., Tschopp J., Karin M. (2006). Intracellular Pattern Recognition Receptors in the Host Response. Nature.

[36] Benko S., Philpott D.J., Girardin S.E. (2008). The Microbial and Danger Signals That Activate Nod-Like Receptors. Cytokine.

[37] Laing K.J., Purcell M.K., Winton J.R., Hansen J.D. (2008). A Genomic View of the Nod-Like Receptor Family in Teleost Fish: Identification of a Novel Nlr Subfamily in Zebrafish. BMC Evol. Biol..

[38] Strober W., Murray P.J., Kitani A., Watanabe T. (2006). Signalling Pathways and Molecular Interactions of *NOD1* and Nod2. Nat. Rev. Immunol..

[39] Correa R.G., Milutinovic S., Reed J.C. (2012). Roles of *NOD1* (Nlrc1) and Nod2 (Nlrc2) in Innate Immunity and Inflammatory Diseases. Biosci. Rep..

[40] Le Bourhis L., Benko S., Girardin S. (2007). *NOD1* and Nod2 in Innate Immunity and Human Inflammatory Disorders. Biochem. Soc. Trans..

[41] Inohara N., Nuñez G. (2003). Nods: Intracellular Proteins Involved in Inflammation and Apoptosis. Nat. Rev. Immunol..

[42] Sha Z., Abernathy J.W., Wang S., Li P., Kucuktas H., Liu H., Peatman E., Liu Z. (2009). Nod-Like Subfamily of the Nucleotide-Binding Domain and Leucine-Rich Repeat Containing Family Receptors and Their Expression in Channel Catfish. Dev. Comp. Immunol..

[43] Li J., Kong L., Gao Y., Wu C., Xu T. (2015). Characterization of Nlr-a Subfamily Members in Miiuy Croaker and Comparative Genomics Revealed Nlrx1 Underwent Duplication and Lose in Actinopterygii. Fish Shellfish Immunol..

[44] Paria A., Makesh M., Chaudhari A., Purushothaman C., Rajendran K. (2018). Nucleotide-Binding Oligomerization Domain-Containing Protein 1 (*NOD1*) in Asian Seabass, *Lates calcarifer*: Cloning, Ontogeny and Expression Analysis Following Bacterial Infection or Ligand Stimulation. Fish Shellfish Immunol..

[45] Chen W., Xu Q., Chang M., Nie P., Peng K. (2010). Molecular Characterization and Expression Analysis of Nuclear Oligomerization Domain Proteins *NOD1* and Nod2 in Grass Carp *Ctenopharyngodon idella*. Fish Shellfish Immunol..

[46] Hou Q.-H., Yi S.-B., Ding X., Zhang H.-X., Sun Y., Zhang Y., Liu X.-C., Lu D.-Q., Lin H.-R. (2012). Differential Expression Analysis of Nuclear Oligomerization Domain Proteins *NOD1* and Nod2 in Orange-Spotted Grouper (*Epinephelus coioides*). Fish Shellfish Immunol..

[47] Gu T., Lu L., Wang J., Tian L., Wei W., Wu X., Xu Q., Chen G. (2018). The *NOD1* and Nod2 in Mandarinfish (*Siniperca chuatsi*): Molecular Characterization, Tissue Distribution, and Expression Analysis. BMC Genet..

[48] Li J., Gao Y., Xu T. (2015). Comparative Genomic and Evolution of Vertebrate *NOD1* and Nod2 Genes and Their Immune Response in Miiuy Croaker. Fish Shellfish Immunol..

[49] Li H., Liu H., Bi L., Liu Y., Jin L., Peng R. (2024). Immunotoxicity of Microplastics in Fish. Fish Shellfish Immunol..

[50] Kim J.G., Lee S.J., Kagnoff M.F. (2004). *NOD1* is an Essential Signal Transducer in Intestinal Epithelial Cells Infected With Bacteria That Avoid Recognition by Toll-Like Receptors. Infect Immun..

[51] Juárez E., Carranza C., Hernández-Sánchez F., Loyola E., Escobedo D., León-Contreras J.C., Hernández-Pando R., Torres M., Sada E. (2014). Nucleotide-Oligomerizing Domain-1 (*NOD1*) Receptor Activation Induces Pro-Inflammatory Responses and Autophagy in Human Alveolar Macrophages. BMC Pulm. Med..

[52] Fritz J.H., Girardin S.E., Fitting C., Werts C., Mengin-Lecreulx D., Caroff M., Cavaillon J.-M., Philpott D.J., Adib-Conquy M. (2005). Synergistic Stimulation Of Human Monocytes And Dendritic Cells By Toll-Like Receptor 4 And *NOD1*- and NOD2-activating Agonists. Eur. J. Immunol..

[53] Petterson T., Jendholm J., Månsson A., Bjartell A., Riesbeck K., Cardell L.-O. (2011). Effects of NOD-like Receptors In Human B Lymphocytes and Crosstalk Between *NOD1*/NOD2 And Toll-Like Receptors. J. Leukoc. Biol..

[54] Mu D., Yang J., Jiang Y., Wang Z., Chen W., Huang J., Zhang Y., Liu Q., Yang D. (2022). Single-Cell Transcriptomic Analysis Reveals Neutrophil as Orchestrator during β-Glucan-Induced Trained Immunity in a Teleost Fish. J. Immunol..

[55] Zhou Z.-Y., Jian Z.-R., Gu J.-L., Jiang X.-Y., He J., Gao Y.-C., Lian S., Bai S.-J., Lu C.-W., Kuang L.-F. (2026). Single-Cell Sequencing Reveals Metabolic Reprogramming and Immune Regulation Underlie The Maintenance Of Teleost Granulomas. Fish Shellfish Immunol..

[56] Hu C., Cui J., He R., Han M., Liu Y., Zhou J., Fan D., Ding X., Xiang L., Lou B. (2025). Single-Cell Profiling Reveals Immune Cell Diversity And Infection-Driven Remodeling In The Spleen Of Marine Teleost *Larimichthys crocea*. Fundam. Res..

[57] Oehlers S.H., Flores M.V., Hall C.J., Swift S., Crosier K.E., Crosier P.S. (2011). The Inflammatory Bowel Disease (Ibd) Susceptibility Genes *NOD1* and Nod2 Have Conserved Anti-Bacterial Roles in Zebrafish. Dis. Model. Mech..

[58] Liu Z., Dong J., Ke X., Yi M., Cao J., Gao F., Wang M., Ye X., Lu M. (2022). Isolation, identification, and pathogenic characteristics of *Nocardia seriolae* in largemouth bass *Micropterus salmoides*. Dis. Aquat. Organ..

[59] Liu W., Deng Y., Tan A., Zhao F., Chang O., Wang F., Lai Y., Huang Z. (2023). Intracellular behavior of *Nocardia seriolae* and its apoptotic effect on RAW264.7 macrophages. Front. Cell. Infect. Microbiol..

[60] Teng J., Li Y., Zhao Y., Zhang Y., Chen D., Liu J., Cui M., Ji X. (2024). Integrated analysis of proteome and transcriptome revealed changes in multiple signaling pathways involved in immunity in the northern snakehead (*Channa argus*) during Nocardia seriolae infection. Front. Cell. Infect. Microbiol..

[61] Inohara N., Ogura Y., Nuñez G. (2002). Nods: A Family of Cytosolic Proteins That Regulate the Host Response to Pathogens. Curr. Opin. Microbiol..

[62] Wang Y., Yang S., Cai X., Huang Z., Tan K., Xu P. (2024). Functional Characterization of *NOD1* from Golden Pompano Trachinotus Ovatus. Fish Shellfish Immunol..

[63] He J., Meng Z., Lu D., Liu X., Lin H. (2021). Recognition of Dap and Activation of NF-κB by Cytosolic Sensor *NOD1* in *Oreochromis niloticus*. Fish Shellfish Immunol..

[64] Li M., Wang Q.-L., Lu Y., Chen S.-L., Li Q., Sha Z.-X. (2012). Expression Profiles of Nods in Channel Catfish (*Ictalurus punctatus*) after Infection with *Edwardsiella tarda*, *Aeromonas hydrophila*, *Streptococcus iniae* and Channel Catfish Hemorrhage Reovirus. Fish Shellfish Immunol..

