# Supplementary figures and images for: Characterization and Immune Function of NOD1 in Snakehead (Channa argus)
Source: Biology (Basel). 2026 Jun 16;15(12):942. doi: 10.3390/biology15120942 (PMC13296148; doi:10.3390/biology15120942)

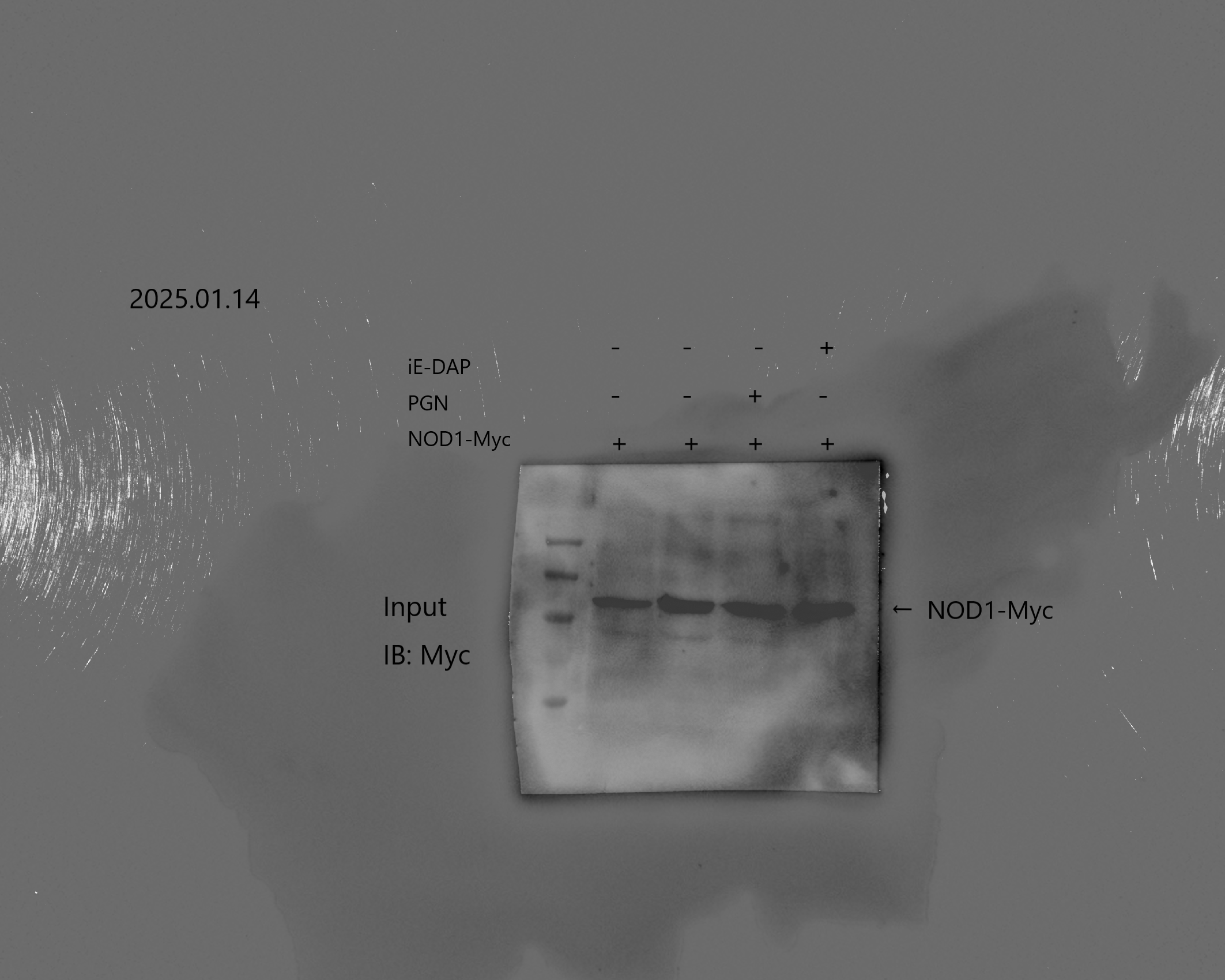

Supplement: Supplementary file 1 [file biology-15-00942-s001.zip › biology-4288725 the full, uncropped original scan of the Western blot image/Input-NOD1.png]

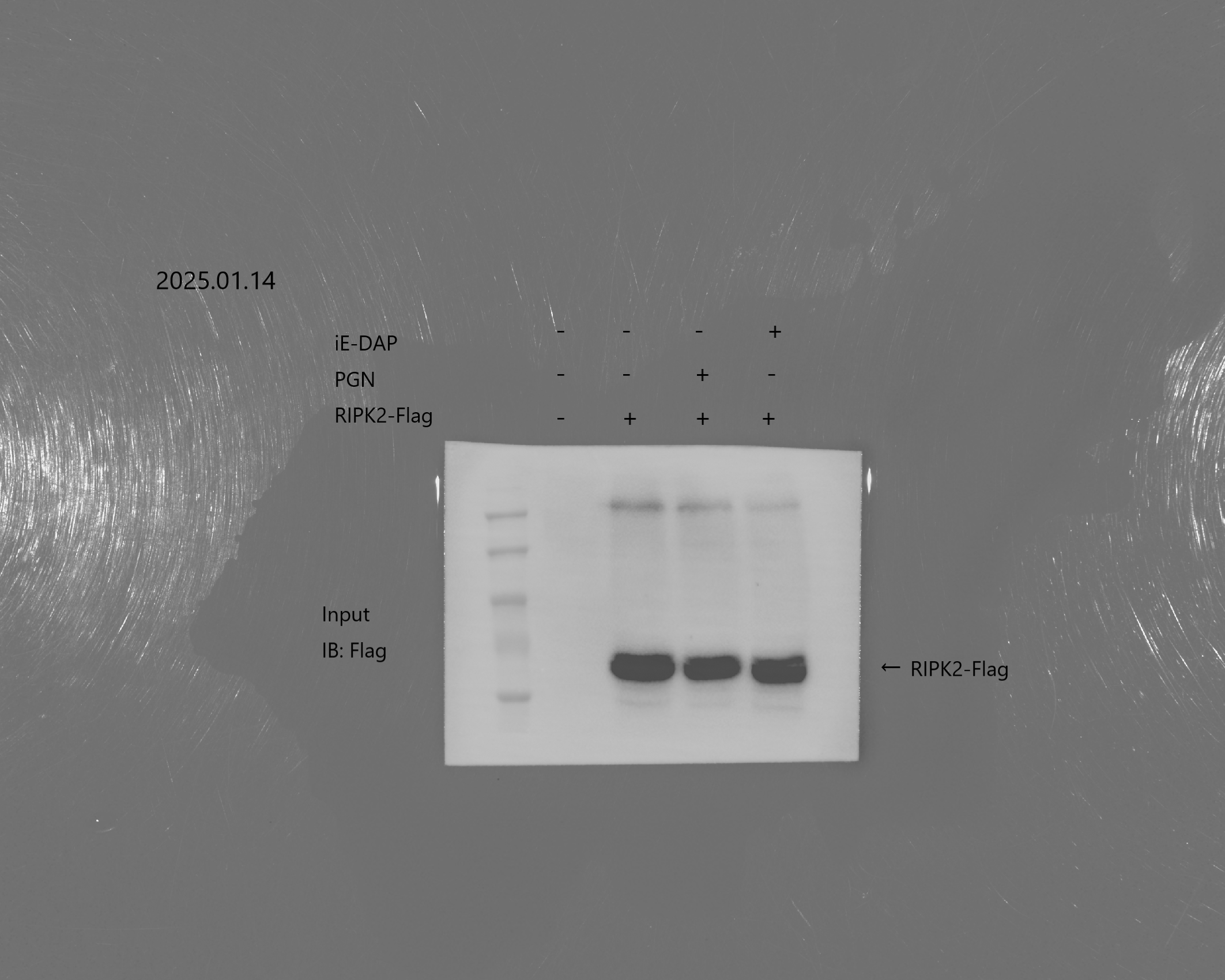

Supplement: Supplementary file 1 [file biology-15-00942-s001.zip › biology-4288725 the full, uncropped original scan of the Western blot image/Input-RIPK2.png]

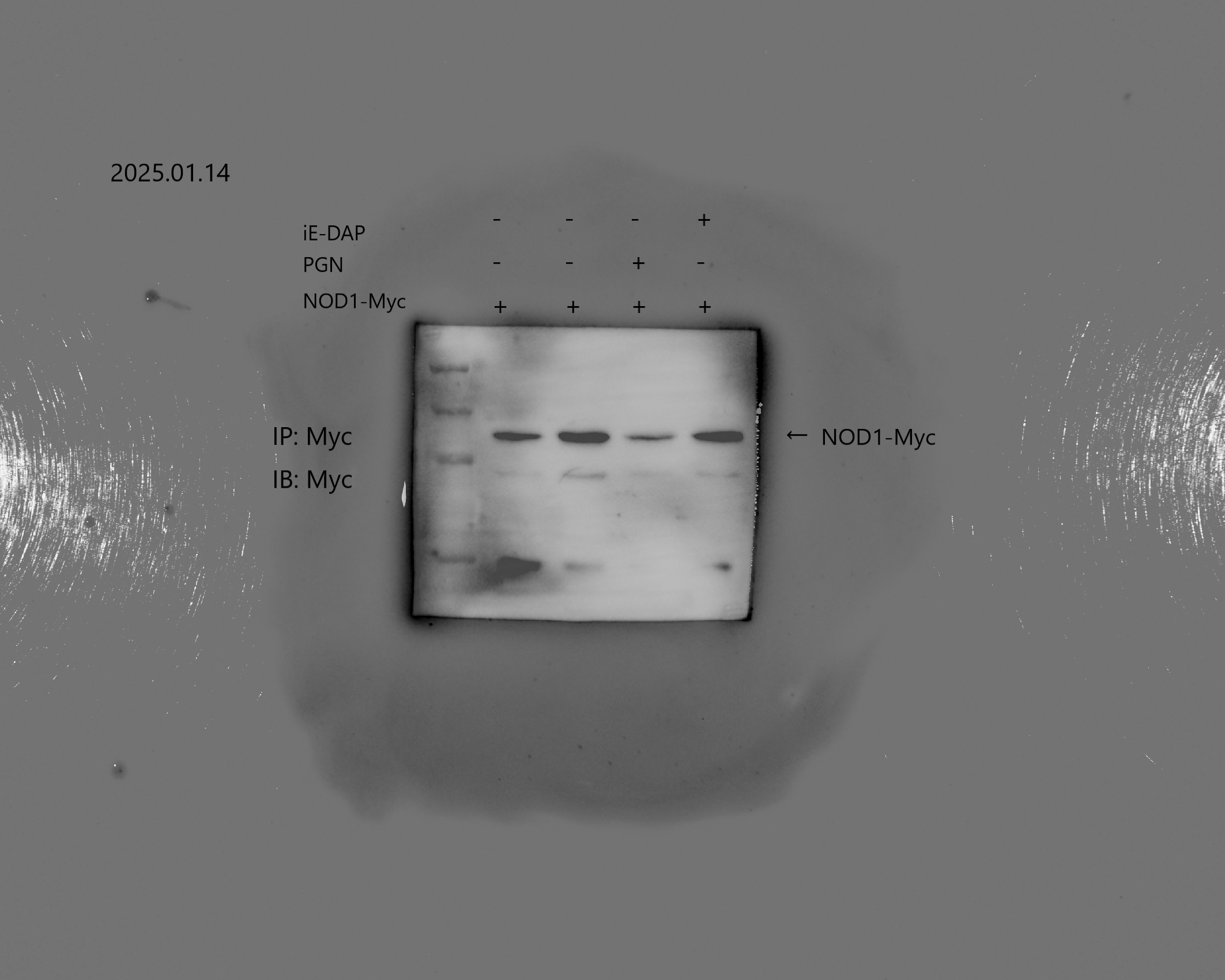

Supplement: Supplementary file 1 [file biology-15-00942-s001.zip › biology-4288725 the full, uncropped original scan of the Western blot image/NOD1.tif]

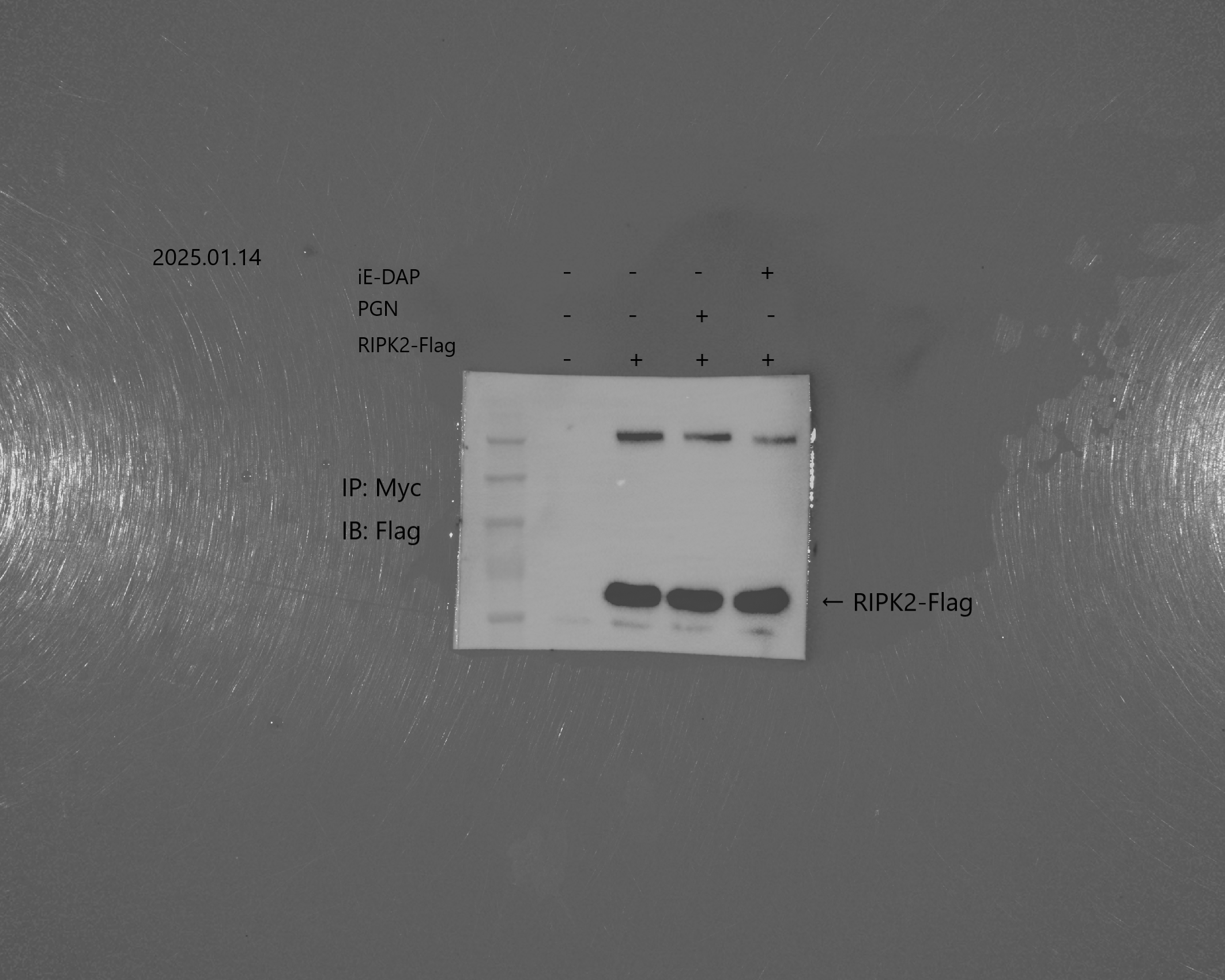

Supplement: Supplementary file 1 [file biology-15-00942-s001.zip › biology-4288725 the full, uncropped original scan of the Western blot image/RIPK2.tiff]
